# *Tetramesa amica* and its parasitoid *Eurytoma amicophaga* (Hymenoptera, Eurytomidae): two new species associated with medusahead, *Taeniatherum caput-medusae* (Poaceae)

**DOI:** 10.3897/zookeys.1005.56353

**Published:** 2020-12-18

**Authors:** Hossein Lotfalizadeh, Jean-Yves Rasplus, Massimo Cristofaro, Francesca Marini

**Affiliations:** 1 Plant Protection Research Department, East Azarbaijan Agricultural and Natural Resources Research & Education Center, AREEO, Tabriz, Iran East Azarbaijan Agricultural and Natural Resources Research & Education Center Tabriz Iran; 2 CBGP, Univ. Montpellier, CIRAD, INRA, IRD, Montpellier SupAgro, Montpellier, France Univ. Montpellier Montpellier France; 3 Centro di Ricerca Casaccia, Agenzia Nazionale Nuove Tecnologie, Energia, e Sviluppo Economico (ENEA), Via Anguillarese, 301, I-00123, Rome, Italy Agenzia Nazionale Nuove Tecnologie Rome Italy; 4 Biotechnological and Biological Control Agency (BBCA) onlus, Rome, Italy Biotechnological and Biological Control Agency Rome Italy

**Keywords:** Biological control, Chalcidoidea, parasitoid, phytophagous, weeds

## Abstract

Medusahead, *Taeniatherum caput-medusae* (Poales: Poaceae), is an annual grass native to central Asia and the Mediterranean region. It is a noxious, invasive weed in much of western North America. During field explorations carried out in Greece in 2017, the new phytophagous eurytomid *Tetramesa amica* Lotfalizadeh, **sp. nov.** and its parasitoid *Eurytoma amicophaga* Lotfalizadeh, **sp. nov.**, also new to science, were recorded for the first time on medusahead. These new species are described and characters that enable to recognize them from their closest relatives are summarized. *Tetramesa* species are generally species-specific gall-inducers. They induce damages that may have a significant impact on the physiology of infested plants by reducing the productivity of flowering heads and seed weight. Based on these data, *T. amica* Lotfalizadeh, **sp. nov.** is currently being investigated as a candidate biological control agent of medusahead.

## Introduction

Medusahead, *Taeniatherum caput-medusae* (L.) Nevski (Poaceae), is a self-pollinating annual grass, native of the Mediterranean region. It has been introduced in northern and north-western Europe, Chile, Australia, as well as in the Americas (Major et al. 1960; Peters 2013; Kyser et al. 2014). This grass is currently listed as a noxious, invasive weed in many states of Western USA, with a 12% spreading rate per year (Rice 2005). In most cases, it becomes quickly established in the localities where it was introduced (Archer 2001). *Taeniatherum caput-medusae* is highly competitive and replaces more desirable annual grasses and forbs (Sharp et al. 1957), but it is almost worthless as forage.

In the past, a few pathogens, such as *Fusarium arthrosporioides*, *Pseudomonas fluorescens*, *Ustilago phrygica* were reported as natural enemies of *T. caput-medusae* (Sforza et al. 2004). A species of eriophyid mite, *Aculodes altamurgiensis*, which is highly specific to medusahead, is currently under investigation as a candidate for biological control (Cristofaro et al. 2020). However, until now no phytophagous insect has been reported to develop on this weed.

Eurytomidae (Hymenoptera, Chalcidoidea) includes 1400–1500 species distributed in 88 genera worldwide (Noyes 2020) and they are mostly parasitoids. In the Palaearctic region, the family includes phytophagous species, mostly in the genera *Tetramesa*, *Bruchophagus* and *Systole*. Most of the 202 described species of *Tetramesa* are known to be species-specific and their host-range is generally restricted to a single grass species, a genus or, in a few cases, on closely related genera (Phillips 1936; Claridge 1961; Dawah 1987). Eggs are laid in the stems of the host plants and the larvae are stem galling and borers, whereas adults feed on nectar (Claridge 1961; Claridge and Dawah 1994; Al-Barrak et al. 2004). Galls induced by the larvae can reduce the productivity of flowering heads and seed weight (Claridge 1961; Spears 1978) and a few *Tetramesa* spp. are sometimes considered pests of crops (Phillips 1927; Spears 1978; Spears and Barr 1985). Claridge (1958, 1961), Szelényi (1968), and Zerova (1965, 1967, 1976, 1978) extensively revised the Palaearctic species of *Tetramesa*, and Graham (1974) studied the species fauna of England.

The significant impact on their host and their high host-specificity make *Tetramesa species* interesting candidates for biological control of weeds. Some species of *Tetramesa* have already been used against invasive grasses such as *Arundo donax* in the USA (Goolsby and Moran 2009) and *Sporobolus* spp. in Australia (Witt and McConnachie 2003).

Until now and despite numerous surveys, no *Tetramesa* has been found associated with the genus *Taeniatherum* (Noyes 2020). Our study presents the first record of a phytophagous eurytomid wasps associated with *T. caput-medusae*. We describe *Tetramesa amica* Lotfalizadeh, sp. nov. and its parasitoid, *Eurytoma amicophaga* Lotfalizadeh, sp. nov. (Hymenoptera: Eurytomidae).

## Materials and methods

Infested samples of *T. caput-medusae* were collected near the town Alexandroupoli (Greece) close to Greek-Turkish border, from 2017 to 2019 and examined in the laboratory. The site was visited once a month, from May to July, and stem galls were collected. Insects were obtained by natural emergence to adults from spikes kept under controlled conditions (24–26 °C, 80% RH, 16L:12D), or by dissecting dry stem galls. Specimens were desiccated using HMDS (Heraty and Hawks 1998) and glued on point cards. Terminology follows Harris (1979) for cuticular sculpture and Lotfalizadeh et al. (2007) for morphology.

The following keys were used to identify *Tetramesa* species: Claridge (1958, 1961) and Zerova (1965, 1967, 1976, 1978). Identification of *Eurytoma* species was performed using keys by Zerova (1976, 1977, 1978, 2010). Images were performed with a Keyence digital microscope (VHX-5000) and were edited in Adobe Photoshop CS6 software. Holotype and paratypes are deposited at **HMIM** (Hayk Mirzayans Insect Museum, Tehran, Iran) and paratypes at **CBGP** (Centre de Biologie pour la Gestion des Populations, Montferrier-sur-Lez, France).

Abbreviations used in the text:

**C1–3** ﬁrst to third clavomere;

**F1, F2, etc.** ﬁrst funiculars, second funiculars, etc.;

**Gt1-n** Gastral terga 1-n;

**OOL** ocular–ocellar line (= the shortest distance between posterior ocellus and adjacent eye margin);

**POL** posterior ocellar line (= the shortest distance between the posterior ocelli).

## Results

Two eurytomid species belonging to *Tetramesa* and *Eurytoma* were obtained from stem galls on *T. caput-medusae*. These two species appeared to be new and are described hereafter

### Tetramesa amica

Taxon classificationAnimaliaHymenopteraEurytomidae

Lotfalizadeh sp. nov.

A2C2F190-DE84-52DB-90E3-72D88FDD14C2

http://zoobank.org/A116AC65-D628-4F3D-AB44-B3766C9F1DB7

Figures 1 , 2

#### Type material.

***Holotype***: female, ex *Taeniatherum caput-medusae*, 8 May 2017, 27 July 2018, and 21 May 2019 (galls collection dates), by F. Marini (deposited in HMIM); Paratypes: 20♀♀ & 3♂♂, same data as holotype (deposited in HMIM & CBGP).

#### Type locality.

Highway E90, between E0 Ardaniou Orestiadas and E0 Alexandroupoli Kipon, ca. 5 km west of the border of Greece-Turkey and 1.3 km northeast of Vrysoùla (40°56'58"N, 26°14'59"E), 40 m above sea level, Dimos Alexandroupoli, Greece.

#### Diagnosis.

*Tetramesa amica* Lotfalizadeh, sp. nov. differs from other species of *Tetramesa* by the combination of the following characters: in female, F1–2 longer than broad, F3–5 as long as broad; fore wing with an obscure black spot under marginal vein; gaster longer than head+ mesosoma; marginal vein much longer than postmarginal and stigmal veins; in male all funiculars longer than wide, with long setae, longer than width of funicule; F1–3 as same as long.

**Figure 1.**
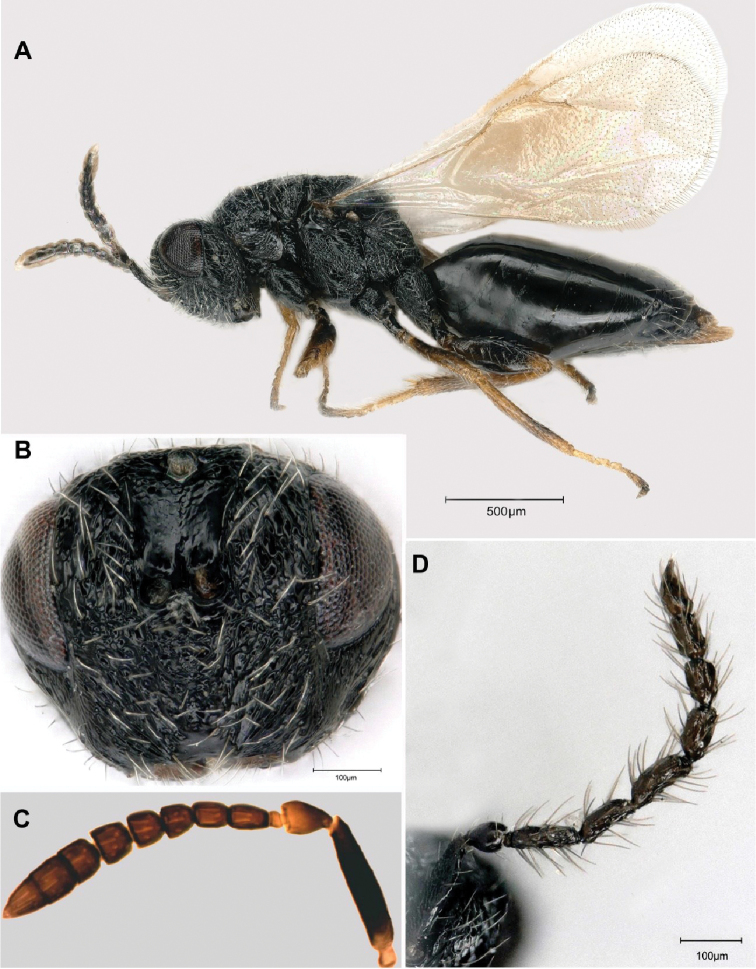
*Tetramesa amica* Lotfalizadeh, sp. nov. **A** female habitus in lateral view **B** head of female in frontal view **C** female antenna **D** male antenna.

#### Description.

**Holotype Female. *Body*** length 2.4 mm. Black, coxae black, pro- and mesofemur brown with a median dark band, metafemur dark brown at apex, all tibiae brown with a faint dark brown median band, tarsi bright yellow, except last tarsomere; tegula dark medially and brown in margin; pronotum with pair of small yellow spots antero-laterally; fore wing hyaline, slightly infuscate below marginal vein; veins yellowish brown. Antenna mainly dark, except scape basally, pedicel in distal half and anellus brownish; ovipositor brown. Setae on body whitish, those on wings blackish.

***Head*** in dorsal view stout, 1.7 × as broad as long, distinctly wider than pronotum; temple rounded laterally, very short, 2.0 × shorter than eye. POL 2.1 × OOL (13:6). Head in frontal view, wider than height (18:14); malar space shorter than longitudinal eye diameter (6:8). Ventral margin of clypeus slightly emarginated (Fig. 1B), dorsally smooth, laterally strigose. Scrobe relatively deep, unclearly bordered, with subparallel edges, tapering only slightly basally. Eye glabrous; cheek shorter than longitudinal diameter of eye (60:80). Face sculpture distinct, reticulated-cellular, with very short and sparse pubescence. Head, in lateral view, ca. 1.3 × as long as wide.

***Antenna*** (Fig. 1C) inserted distinctly above middle of face; scape long (100:22), not convex, reaching level of anterior ocellus; pedicel 1.36 × as long as wide (34:25); with 5 funiculars, anellus small, ca. 1.5× as broad as long (8:14); F1 long, 1.5 × as long as wide (32:16), F2 longer than broad (27:20), F3 as long as broad (24:24), F4 slightly wider (24:25), F5 wider than long (25:28); with three clavomeres, clavomeres clearly separated, C1 (24: 30), C2 (27:30), C3 (33:30), width of clava exceeding width of flagellum (30:28).

**Figure 2.**
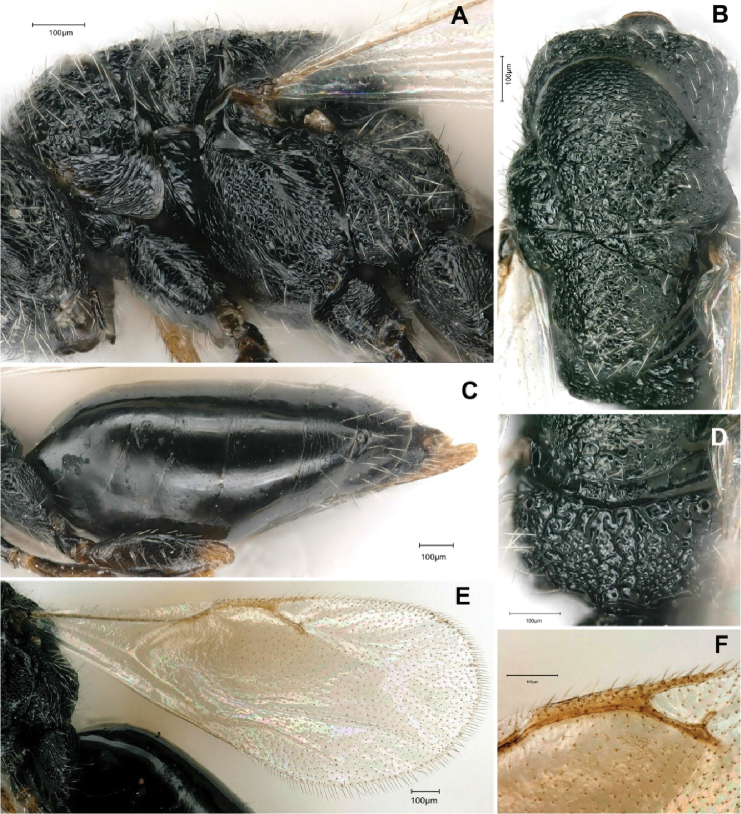
*Tetramesa amica* Lotfalizadeh, sp. nov., female **A** mesosoma in lateral view **B** mesosoma in dorsal view **C** metasoma in lateral view **D** propodeum in dorsal view **E** fore wing **F** fore wing venation.

***Mesosoma*** in lateral view elongated (Fig. 2A), relatively convex, with mesonotum and mesoscutellum at the same level. Propodeum 2.0 × as wide as long (14:7), with slight median depression, three longitudinally irregular rugae, peripherally coarsely rugose (Fig. 2D), slightly inclined relative to mesonotum (ca. 70°), in dorsal view ca. 0.7 × as long as mesoscutellum (70:105), almost half as long as mesoscutum (70:140). Mesoscutellum as long as wide medially. Pronotum and mesonotum reticulate, with scattered and inconspicuous umbilicate sculpture, more distinct on mesoscutellum and pronotum. Metacoxa elongate, weakly reticulated.

***Fore wing*** (Fig. 2E) ca. 2.2 × as long as its maximum width, infuscate under marginal vein. Marginal vein relatively long and slightly expanded; ratio of marginal, postmarginal and stigmal veins: 70:55:55 (Fig. 2F).

***Metasoma*** elongated, narrowed apically (in lateral view) (Fig. 2C), longer than head + mesosoma, with extremely short petiole, Gt1 longest, shorter than Gt2 and Gt3 combined; relative measurements Gt1–7: 26, 23, 17, 10, 13, 5, 7. All terga shiny, Gt5–7 weakly reticulated.

**Male.** Length of body 2.1–2.3 mm. Coloration and sculpture as in females, but yellow spots smaller and predominant on face and upper corners of pronotum. Antenna (Fig. 1D) with seven flagellomeres and long pubescence. Petiole of first tergum short, at most twice longer than its width. Metasoma long, 0.5–0.65 × as long as mesosoma.

#### Comparative notes.

*Tetramesa amica* is closely related to *Tetramesa inermis* Erdös, 1963, *T. matrana* Erdös, 1969, and *T. cylindrica*. Diagnostic characters that enable one to discriminate *T. amica* sp. nov. from these species are presented in Tables 1–3.

The antenna of *T. amica* sp. nov. resembles that of *T. fumipennis* except F1 that is not constricted basally (Fig. 1C) (conical in *T. fumipennis*), with five funiculars, with three clavomeres (respectively six and two in *T. fumipennis*), head in its lower part wider than in *T. fumipennis* and gaster more flattened dorsally than in *T. fumipennis*.

**Table 1.** Features distinguishing *Tetramesa amica* Lotfalizadeh, sp. nov. from *Tetramesa inermis* Erdös, 1963.

Characters	*Tetramesa amica* Lotfalizadeh, sp. nov.	*Tetramesa inermis* Erdös, 1963†
Pronotal antero-lateral yellow spots	With a pair of small yellow spots, hardly seen dorsally	With a pair of relatively large spots, well seen dorsally
Frons sculpture in the lower part	Laterally straight and medially smooth (Fig. 1B)	Entirely straight
Antennal anellus in female	Wider than long (Fig. 1C)	Longer than wide
Length of funiculars in female	F1 ca. 1.5 × as long as wide, F2 longer than broad, F3–5 as long as broad (Fig. 1C)	F1 ca. 1.5 × as long as wide, F2–3 square, F4–5 transverse
Length of clava in female	Longer than the three pre-claval funiculars together (83:72) (Fig. 1C)	Equal to the three pre-claval funiculars together
Male antenna	Funicule thick, funiculars constricted basally and apically (Fig. 1D)	Funicule filiform, funiculars without basal and apical constriction
Sculpture of mesoscutellum	Identical to pronotum (Fig. 2B)	Coarser than pronotum
Uncus of stigma	Distinct and long (Fig. 2F)	As usual (not especially long)
Host plant	*Taeniatherum caput-medusae*	*Bromus* spp.

† See figures in Erdös (1963) and Zerova (1976).

**Table 2.** Features distinguishing *Tetramesa amica* Lotfalizadeh, sp. nov. from *Tetramesa matrana* Erdös, 1969.

Characters	*Tetramesa amica* Lotfalizadeh, sp. nov.	*Tetramesa matrana* Erdös, 1969
Funiculars in female	F2 longer than broad, F3–5 as long as broad (Fig. 1C)	F2–3 as long as broad, F4–5 transverse
Length of clava	Longer than the three pre-claval funiculars together (83:72) (Fig. 1C)	Equal to the three pre-claval funiculars together
Sculpture of mesoscutellum	Identical to pronotum (Fig. 2B)	Coarser than pronotum.
Propodeum	coarsely rugose (Fig. 2D)	almost non-sloping, highly shiny, densely reticulate
Host plant	*Taeniatherum caput-medusae*	*Arrhenathrerum elatius* L.

**Table 3.** Features distinguishing *Tetramesa amica* Lotfalizadeh, sp. nov. from *Tetramesa cylindrica* (Schlechtendal, 1891).

Characters	*Tetramesa amica* Lotfalizadeh, sp. nov.	*Tetramesa cylindrica* (Schelechtendal, 1891)†
Width of the head (frontal view)	1.2 × wider than long (Fig. 1B)	0.8 × wider than long
Length of funiculars of the female	F1–2 longer than wide, F3–5 quadrate (Fig. 1C)	Only F1 longer than wide, F2–5 quadrate
Male antenna	Funiculars non-depressed medially (Fig. 1D)	F2–4 depressed medially
Gastral sculpture	Mainly smooth (Fig. 2C)	Finely alutaceous dorsally
Length of metasoma	1.1 × as long as mesosoma + head (Fig. 1A)	As long as mesosoma + head
Postmarginal vein	1.4 × the length of marginal vein (Fig. 2F)	As long as marginal vein
Stigma vein	As long as postmarginal vein (Fig. 2F)	0.7 × the length of postmarginal vein
Host plant	*Taeniatherum caput-medusae*	*Stipa capillata*

† See figures in Zerova (1965, 1967).

#### Etymology.

The specific epithet derives from the Latin noun *amicus* (i.e., friendship) and refers to the friendship between entomologists from different countries (France, Iran, and Italy), which made possible the sampling, discovery, and description of this new species.

#### Host.

Medusahead, *Taeniatherum caput-medusae* (L.) Nevski (Poaceae). Adults are phytophagous and lay eggs into medusahead stems. Oviposition and larval development induce a response of the plant, which produces stem galls, from which adults emerge.

### Eurytoma amicophaga

Taxon classificationAnimaliaHymenopteraEurytomidae

Lotfalizadeh sp. nov.

574CFBE3-C74A-59B6-B25B-568D713DB5D5

http://zoobank.org/1FB6F92C-A988-4BCE-AEEB-3FD6627F0FFE

Figures 3 , 4

#### Type material.

***Holotype***: *female*, ex *Tetramesa amica* Lotfalizadeh, sp. nov. on *Taeniatherum caput-medusae*, 28 May 2017, 27 July 2018, and 21 May 2019 (galls collection dates), F. Marini leg. (deposited in HMIM). ***Paratypes***: same data as holotype, 1♀ & 5♂♂ (deposited in HMIM & CBGP).

**Figure 3.**
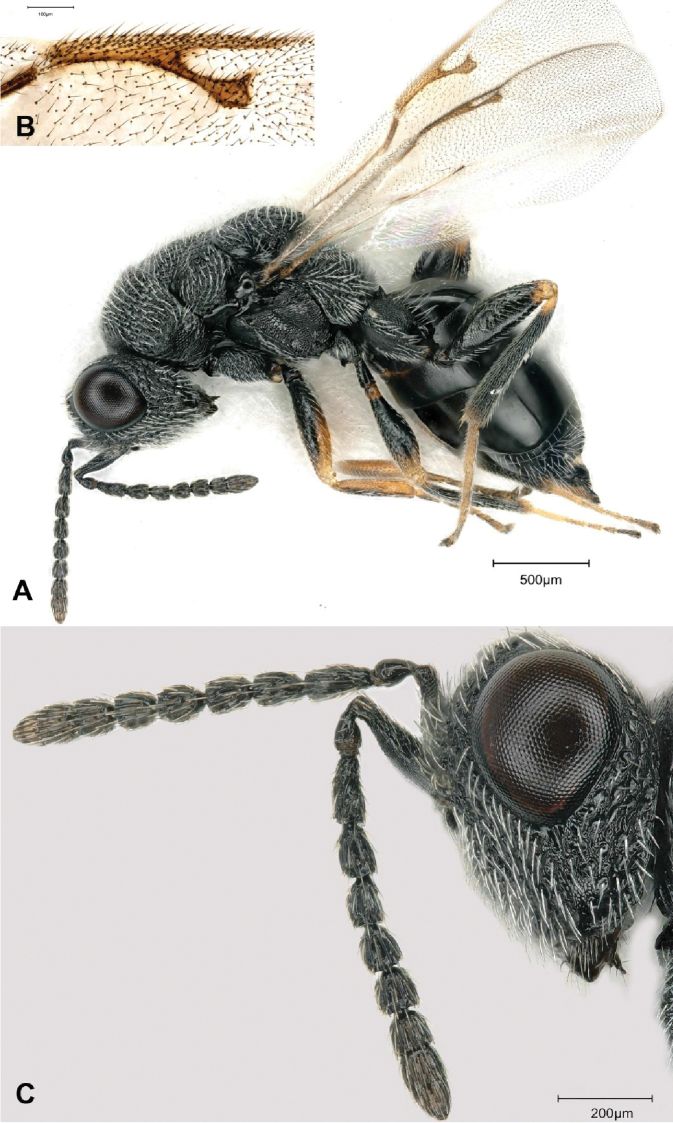
*Eurytoma amicophaga* Lotfalizadeh, sp. nov., female **A** female habitus in lateral view **B** fore wing venation **C** head and antennae in lateral view.

#### Type locality.

Highway E90, between E0 Ardaniou Orestiadas and E0 Alexandroupoli Kipon, ca. 5 km west of the border of Greece-Turkey and 1.3 km northeast of Vrysoùla (40°56'58"N, 26°14'59"E), 40 m above sea level, Dimos Alexandroupoli, Greece.

#### Diagnosis.

**Figure 4.**
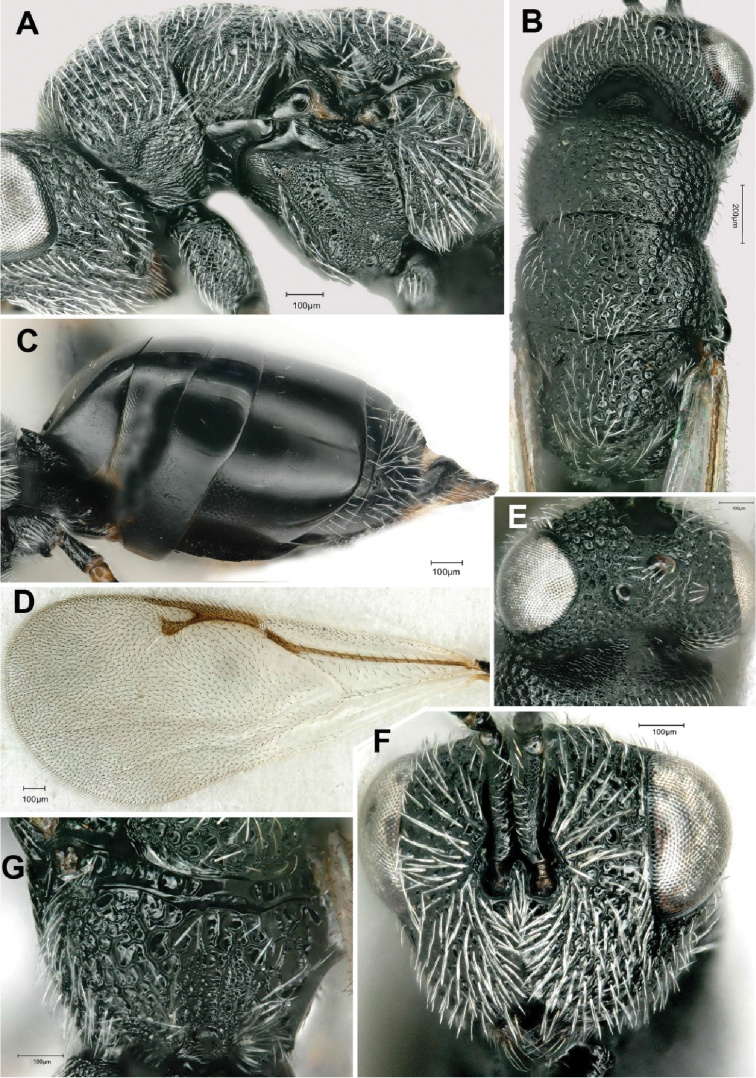
*Eurytoma amicophaga* Lotfalizadeh, sp. nov., female **A** mesosoma in lateral view **B** mesosoma in dorsal view **C** metasoma in lateral view **D** fore wing **E** head in dorsal view **F** head in frontal view **G** propodeum in dorsal view.

All funiculars longer than broad, with F1 ca. 2.5 × as long as wide (Fig. 3A). Pro- and mesonotum densely punctured (Fig. 4B), and narrow interspaces coriaceous sculpture. Gaster long, as long as mesosoma + head. Gt4 longest tergum, ovipositor horizontal.

#### Description.

**Holotype. Female. *Body*** length 3.3 mm. Coloration: body black; following areas yellow to reddish brown: profemur apically and interiorly, protibia interiorly, mid femur and tibia basally and apically, metafemur apically and metatibia basally, three basal tarsomeres, distal spurs of tibiae; wing veins brown. Valvulae mostly dark brown.

***Head*** 1.3 × as wide as long (164:125) (Fig. 4F). Relative measurements: head width 158, head length 130, width of frontovertex 100, length of eye 62, length of temple 12, ocellar diameter 15, distance between lateral and median ocelli 18, POL 35, OOL 20, malar space 45, height of eye 72. Head relatively transverse in dorsal view (140:85) (Fig. 4E). Anterior outline of frons slightly convex. Temple with straight lateral outline, hardly converging back-wards and strongly angulate with occiput. Clypeus hardly emarginated. Lower face mostly strigose laterally, ridges not reaching antennal toruli above, face punctured latero-dorsally (Fig. 3C). Frons covered with piliferous punctures. Malar carina raised near oral fossa, curved, incomplete, not reaching lower margin of eye above (Fig. 3C). Gena entirely punctured, inter-punctures finely reticulate, gena without area of fine sculpture behind malar carina. Genal carina raised; outline of carina forming blunt angle above oral fossa. Inter-torular space deeply sulcate, bearing one row of hairs. Inner margins of antennal toruli raised. Lateral margin of antennal scrobes carinate, forming a raised lobe above toruli. Postgenal laminae expanded, visible in lateral view as a small tooth (Fig. 3C). Scape 55, slightly swelling ventrally, straight dorsally. Pedicel + flagellum as long as width of head (130). Pedicel short, 2 × as long as wide (20:10) with a basal bottleneck (Fig. 3C). With five funiculars, all funiculars longer than wide, F4–5 as long as broad (Fig. 3C). F1 longer than pedicel (25:20) (Fig. 3C), 2.5 × as long as wide (25:10), following segments progressively decreasing in length (20, 17, 17, 15, 15). With three clavomeres (38), slightly tapering to apex, and narrowly rounded (Fig. 3C).

Relative measurements of mesosoma: length 205, width 120, length of pronotal collar 105, mesoscutum as long as mesoscutellum70; width of mesoscutellum 75. Pro- and mesonotum densely punctured (Fig. 4B), inter-punctures coriaceous. Notauli impressed but obliterated by sculpture of mesoscutum, especially in posterior part. Axillar grooves obliterated by sculpture anteriorly, not reaching transscutal line. Dorsal outline of mesoscutellum strongly convex. Frenal arms visible laterally. Propodeum slightly sloping, slightly inclined with main axis of mesonotum (Fig. 4D), broadly concave in middle, with an areolate median groove, not delimited by submedian ridges and visible through change in sculpture only, generally reticulate-areolate. Adscrobal carina of mesopleuron distinctly raised ventrally (Fig. 4A); femoral depression mostly reticulate, with some carinulae. Mesepimeron mostly reticulate ventrally, striolate dorsally, with usual longitudinal rugae originating from its posterior margin, finely reticulate ventrally. Procoxae with usual oblique groove and S-like basal ridge of *Eurytoma*. Mesocoxae with well-developed lamella distally, striolate on anterodorsal surface. Metacoxa entirely reticulate, bare dorsally at base. Fore wing ca. 2.3 × longer than wide (175:75) (Fig. 4D), marginal vein 1.2 × as long as stigmal vein (80:65); postmarginal vein (75) slightly shorter than marginal vein (Fig. 3B). Basal cell partly sparsely hairy; speculum reduced to a narrow stripe behind parastigma; dorsal surface of costal cell with three or four rows of setae.

***Petiole*.** Gastral petiole transverse, bearing usual dorso-median and lateral teeth, which are acute. Gaster longer than mesosoma (105:90) (Fig. 4C), height 48, respective lengths of Gt1–6 on median line as 26, 15, 12, 24, 18 and 28; syntergum 30; maximal lateral length of Gt4: 75. Gt1 with usual basal submedian pits. Posterior margins of Gt5 diverging ventrally, margin of Gt4 convex dorsally. Gt2 and Gt3 basally (in lateral view) and Gt4 ventrally with a well delimited area showing reticulate sculpture. Gt4 not completely overlapping Gt5 laterally and emarginate on posterior margin dorsally. Gt5 not punctulate dorsally. Gt6 not carinate dorsally. Valvulae not ascending backwards with main axis of gaster (Fig. 4C).

**Figure 5.**
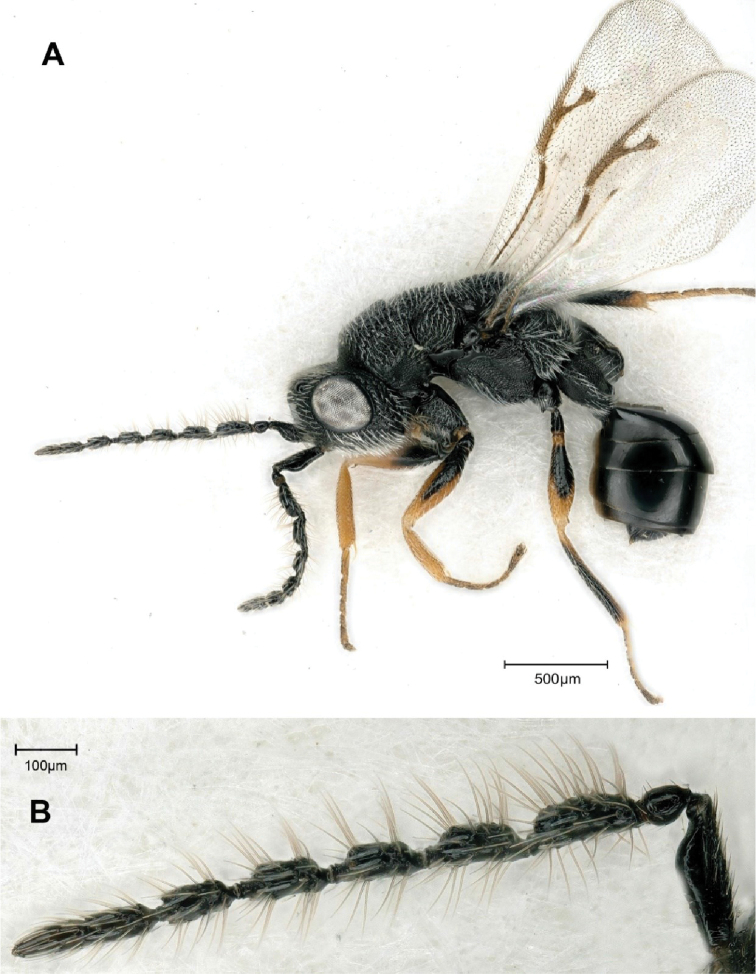
*Eurytoma amicophaga* Lotfalizadeh, sp. nov., male **A** male in lateral habitus, **B** antenna.

**Male** (Fig. 5A). Body length 1.6 mm. Characters distinctive from female: Scape distinctly swollen anteriorly and ventrally (Fig. 5B). With 7 flagellomeres, basally wider and longer than distal, segments pedunculate with at least 2 rows of erect setae on F2–F5 and ca. 1.5–2 × as long as wide, last two flagellomeres definitely separated. Relative measurements of scape 75:25, of pedicel 28:27. Gastral petiole elongate, as long as metacoxa, evenly reticulate, cylindrical in lateral view, lateral length ca. 1.4× as long as greatest width, with slight ventral carina (Fig. 5A).

#### Variations.

Body length ranges from 2.5 to 3.6 mm. Pro and mesofemora, scape sometimes nearly entirely black. Marginal vein slightly to distinctly longer than stigmal vein.

#### Comparative notes.

*Eurytoma amicophaga* Lotfalizadeh, sp. nov. is distinct from other species of this species group. It is characterized by elongated funiculars, although *E. steffani* Claridge, 1959 and *E. pollux* Claridge, 1959 share similar funicular segments. However, *E. steffani* has all funicular segments longer than broad (F4–5 quadrate in *E. amicophaga* Lotfalizadeh, sp. nov.). *Eurytoma pollux* obviously differs from *E. amicophaga* in the longer head in frontal view, less than 1.2 × longer than broad (wider head, more than 1.3 × longer than broad in *E. amicophaga* Lotfalizadeh, sp. nov.) and marginal vein more than 1.5 × as long as stigmal vein (less than 1.5 × as long as stigmal vein in *E. amicophaga* Lotfalizadeh, sp. nov.). *Eurytoma amicophaga* Lotfalizadeh, sp. nov. is also closely related to *E. festucae* Zerova, 1977 and may be separated by characters summarized in Table 4.

**Table 4.** Features distinguishing *Eurytoma amicophaga* Lotfalizadeh, sp. nov. from *Eurytoma festucae* Zerova, 1977.

Characters	*Eurytoma amicophaga* Lotfalizadeh, sp. nov.	*Eurytoma festucae* Zerova, 1977†
Width of head (frontal view)	1.2 × as wide as long (Fig. 4F)	1.9 × as wide as long
Male antenna	Funiculars long, F1 more than 2 × as long as wide (Fig. 5B)	Funiculars short, F1 distinctly < 2 × as long as wide
Scape in male antenna	long, 2.8 × as long as wide	short, 2.2 × as long as wide
F1 length	2.5 × as long as wide (Fig. 3A)	2 × as long as wide
Marginal vein	Long, more than 1.5 × as long as stigmal vein (Fig. 3A)	Short, as long as stigmal vein
Host	*Tetramesa amica* Lotfalizadeh, sp. nov. on *Taeniatherum caput-medusae*	*Tetramesa brevicollis* on *Festuca* spp.

† See figures in Zerova (2010).

#### Etymology.

The specific name refers to the host species (*Tetramesa amica* Lotfalizadeh, sp. nov.) with which holotype is associated.

#### Host.

*Tetramesa amica* Lotfalizadeh, sp. nov. (Hymenoptera: Eurytomidae). Larvae feed on *T. amica* larvae and adults emerge from the stem galls caused by *T. amica* larvae on medusahead plants.

## Discussion

Several studies have been carried out on the taxonomy and biology of species of *Eurytoma* and *Tetramesa* associated with grasses in the Palaearctic region. However, no revision of these genera has been published so far and the identification of species remains difficult. This is also due to the rather uniform morphology of these wasps that renders their identification challenging (Henneicke et al. 1992; Lotfalizadeh et al. 2007). *Tetramesa amica* Lotfalizadeh, sp. nov. belongs to the *cylindrica* species group of *Tetramesa*. This distinctive group of species is characterized by the alutaceous sculpture of head and thorax, without distinct umbilicate punctures, and with small pronotal yellow spots (Claridge 1961). The *cylindrica* species group includes *T. aciculata* (Schlechtendal, 1891), *T. cylindrica* (Schlechtendal, 1891), *T. dispar* Zerova, 1965, *T. ukrainica* Zerova, 1965, *T. punctata* Zerova, 1965 and *T. scheppigi* (Schlechtendal, 1891) (Claridge 1961; Zerova 1976).

Several species of *Tetramesa* have been shown to efficiently affect the populations of their host plants. Substantial reduction in seed weight was reported for an undescribed *Tetramesa* on *Aristida longiseta* Steud., *Sitanion hystrix* (Nutt.), *Sporobolus cryptandrus* (Torr.) and *Stipa comata* Trin. & Rupr. (i.e., 47, 33, 46, and 60%, respectively), with consequent reduction in seed germination (e.g., up to 99% of *A. longiseta* seeds not germinating) (Spears and Barr 1985). Witt and Mc Connachie (2003) collected a stem-boring *Tetramesa* species on *Sporobolus pyramidalis* P. Beauv, *S. africanus* Poir. A. Robyns and Tournay and *S. natalensis* (Steud.) in South Africa. They reported a high rate of prevalence of *Tetramesa* in stems with up to 33% of *S. pyramidalis* infested by *Tetramesa* larvae. Inflorescences of approximately 60% of the infested culms were malformed and significantly shorter than non-infested one. Finally, the stem-galling wasp *T. romana* is considered one of the best biological control agents released in USA to control giant reed (*Arundo donax*) (Goolsby and Moran 2009; Goolsby et al. 2016; Moran et al. 2017). Therefore, based on our current knowledge on *Tetramesa* spp., *T. amica* exhibits characteristics to be considered a prospective biocontrol agent against *T. caput-medusae*. Since few biological and ecological informations are currently available on this phytophagous species, more studies are needed to better characterize biological traits, host specificity, duration of immature stages, number of generations, fecundity, and longevity of adults. More information is also needed on its natural distribution in the Western Palaearctic region.

Species of *Tetramesa* are frequently parasitized by other chalcid wasps or exploited by inquilines. These antagonistic species appear to be also highly specialized on one or a few host species (Dawah et al. 1995, 2002; Dubbert et al. 1998; Matsumoto and Saigusa 2001). During our field surveys, we discovered that *T. amica* is parasitized by *E. amicophaga* Lotfalizadeh, sp. nov. Females of this species exhibits fusiform ﬂagellomeres (Fig. 3C), a relatively long marginal vein (1.2 × as long as stigmal vein) (Fig. 3B), and a horizontal ovipositor (Fig. 4C) which indicate that *Eurytoma amicophaga* Lotfalizadeh, sp. nov. belongs to the *appendigaster* species group as outlined in Claridge (1959) and Lotfalizadeh et al. (2007) (named the *phragmiticola* species group by Zerova (2010)). This species group contains parasitoids of *Tetramesa* species developing in grass stems. The exact biology of this parasitoid remains to be discovered, and studies are requested to better evaluate parasitism rates of *E. amicophaga* and how it may affect the performance of *T. amica* to control medusahead.

## Supplementary Material

XML Treatment for Tetramesa amica

XML Treatment for Eurytoma amicophaga
